# Preliminary evidence that ketamine alters anterior cingulate resting-state functional connectivity in depressed individuals

**DOI:** 10.1038/s41398-023-02674-1

**Published:** 2023-12-01

**Authors:** Laith Alexander, Peter C. T. Hawkins, Jennifer W. Evans, Mitul A. Mehta, Carlos A. Zarate

**Affiliations:** 1 Institute of Psychiatry, Psychology and Neuroscience, King’s College London & Centre for Neuroimaging Sciences, King’s College London, London, UK; 2 Experimental Therapeutics and Pathophysiology Branch, National Institute of Mental Health, Bethesda, MD USA

**Keywords:** Depression, Clinical pharmacology, Neuroscience

## Abstract

Activity changes within the anterior cingulate cortex (ACC) are implicated in the antidepressant effects of ketamine, but the ACC is cytoarchitectonically and functionally heterogeneous and ketamine’s effects may be subregion specific. In the context of a double-blind randomized placebo-controlled crossover trial investigating the clinical and resting-state fMRI effects of intravenous ketamine *vs.* placebo in patients with treatment resistant depression (TRD) *vs.* healthy volunteers (HV), we used seed-based resting-state functional connectivity (rsFC) analyses to determine differential changes in subgenual ACC (sgACC), perigenual ACC (pgACC) and dorsal ACC (dACC) rsFC two days post-infusion. Across cingulate subregions, ketamine differentially modulated rsFC to the right insula and anterior ventromedial prefrontal cortex, compared to placebo, in TRD *vs.* HV; changes to pgACC-insula connectivity correlated with improvements in depression scores. Post-hoc analysis of each cingulate subregion separately revealed differential modulation of sgACC-hippocampal, sgACC-vmPFC, pgACC-posterior cingulate, and dACC-supramarginal gyrus connectivity. By comparing rsFC changes following ketamine *vs.* placebo in the TRD group alone, we found that sgACC rsFC was most substantially modulated by ketamine *vs.* placebo. Changes to sgACC-pgACC, sgACC-ventral striatal, and sgACC-dACC connectivity correlated with improvements in anhedonia symptoms. This preliminary evidence suggests that accurate segmentation of the ACC is needed to understand the precise effects of ketamine’s antidepressant and anti-anhedonic action.

## Introduction

Ketamine is a rapidly acting antidepressant drug, with promising effects in patients with treatment-resistant depression (TRD). Ketamine is also effective in ameliorating anhedonia, which can otherwise be refractory to conventional antidepressant treatment [1–3]. Both preclinical and clinical evidence suggests that modulation of anterior cingulate cortex (ACC) activity is important in ketamine’s antidepressant and anti-anhedonic effects (as reviewed in [4]).

The ACC is heterogenous and is comprised of several subregions including the subgenual (sgACC), perigenual (pgACC), and dorsal (dACC) regions – each of which has different anatomical [5] and functional [6] connectivity. Distinctions have been drawn, for example, between a dorsal dACC-parietal attention network and a ventral pg/sgACC-default mode affective network, suggesting different functions across a small anatomical extent [6]. It is therefore likely that different ACC subregions likely undergo differential modulation following ketamine administration, and these subregions may contribute differently to ketamine’s therapeutic action.

It remains unclear which subregions are important in ketamine’s antidepressant effects. Rodent studies suggest that the rodent homolog of sgACC (specifically Brodmann Area [BA]25) – infralimbic cortex (IL) – is a critical site of action mediating ketamine’s acute antidepressant-like effects [7–9], and the rodent homolog of pgACC – prelimbic cortex (PL) – mediates sustained effects [10–12]. In humans, whilst several neuroimaging studies suggest that ketamine-induced changes in ACC activity or connectivity can correlate with antidepressant effects [13–15], the locus of change varies cross the extent of the ACC [16].

Furthermore, whether certain subregions are more or less important in ketamine’s anti-anhedonic effects is uncertain. Preclinical work in marmosets – whose ACC is more homologous to human ACC – suggests that ketamine’s modulation of sgACC and a downstream reward-related network (including dACC, insula, and ventral striatum) is critical in ameliorating reward-related deficits induced by sgACC over-activity [17, 18]. In humans, work has implicated changes throughout the ACC. In patients with bipolar disorder and in patients with MDD, anti-anhedonic effects of ketamine correlate with increased ^18^F-FDG uptake in dACC [1, 2]. Ketamine has also been shown to modulate pgACC-striatal connectivity differentially in TRD compared to healthy volunteers, correlating with improvements in anhedonia symptoms [19]. Finally, ketamine reduces sgACC hyperactivity to positive incentives on a monetary incentive delay task, again correlating with improvements in anhedonia [20].

In light of this growing body of preclinical and clinical work, circuit-based perspectives on ketamine’s action have emerged, with the ACC as a region of importance [4]. Particular interest has focused on impaired top-down regulation of sgACC by ‘higher’ cortical regions such as dorsolateral PFC (BA9/46), frontopolar/anterior ventromedial prefrontal cortex (vmPFC; BA10m), and pgACC (BA32) [21]. Comprehensive tract tracing work in macaques shows these regions are intimately connected [22]. Top-down regulation of sgACC can be disrupted by chronic stress [23, 24], and successful antidepressant treatment across a variety of modalities may be associated with restoration of top-down inhibition of sgACC by higher prefrontal regions [21, 25].

The modulation of different ACC subregions by ketamine has not been compared in a single study. In the context of a double-blind, randomized placebo-controlled trial, we compared the modulation of sgACC, pgACC and dACC resting-state functional connectivity (rsFC) with the rest of the brain, following ketamine *vs.* placebo, in patients with treatment-resistant depression (TRD) *vs.* healthy volunteers (HV). We additionally investigated how these changes correlated with ketamine’s antidepressant and anti-anhedonic effects. We hypothesized that ketamine would differentially modulate rsFC across sgACC, pgACC, and dACC, and that modulations of ACC rsFC may be relevant to the therapeutic effects of ketamine. Our hypotheses were not pre-registered and were carried out as a post-hoc analysis of a pre-existing dataset (NCT00088699).

## Methods

The methods in this paper are similar to those described previously [19, 26, 27].

### Participants

Data for 50 participants (21 HV and 29 TRD) were taken from a randomized-clinical trial (NCT00088699), with inclusion and exclusion criteria previously published [26, 27]. TRD patients were diagnosed with a current major depressive episode without psychotic features, had not responded to at least one adequate antidepressant trial during their current episode (mean ± SD number of failed lifetime trials: 6 ± 3), and had a Montgomery-Åsberg Depression Rating Scale (MADRS [28]) score of 20 or more at screening and before each infusion. Before scanning, all TRD patients were medication-free for at least two weeks (three weeks for aripiprazole, 5 weeks for fluoxetine). HVs had no Axis I disorder.

Information regarding participant characteristics can be found in Table 1 and in Supplementary Methods (Participant Information). All participants provided written informed consent, and the study was approved by the NIH IRB.

**Table 1:** Participant demographic and clinical characteristics.

Variable	Healthy volunteers (HV)	Treatment-resistant depression (TRD)
Total number in sample	21 (13 female, 8 male)	29 (18 female, 11 male)
Age in years	35 (±11)	36 (±10)
BMI in kg/m^2^	27.8 (±4.6)	26.4 (±5.7)
Length of illness		21 (±11) years
Length of current episode		45 (±73) months
Number of failed treatments		6 (±3) adequate trials
Baseline MADRS score	1 (±2)	33 (±5)
Baseline SHAPS score	18 (±4)	40 (±4)
Baseline TEPS-anticipatory score	47 (±6)	23 (±8)
Baseline TEPS-consummatory score	40 (±5)	23 (±5)
Figs. 1–3 & 4A: Number of post-ketamine fMRI images	20 (12 female, 8 male)	26 (16 female, 10 male)
Figs. 1–3 & 4A: Number of post-placebo fMRI images	17 (11 female, 6 male)	25 (13 female, 12 male)
Fig. 4B, C: Number of subjects with both fMRI and SHAPS/TEPS data, both post-ketamine and post-placebo		11 (5 female, 6 male)
Fig. 5A, B: Number of subjects with fMRI and SHAPS data post-ketamine		15 (9 female, 6 male)
Fig. 5A: Number of subjects with fMRI and SHAPS data post-placebo		12 (6 female, 6 male)

(± standard deviation, where relevant).

### Study procedures

Resting-state (rs)fMRI imaging was carried out in the context of a double-blind randomized placebo-controlled crossover study. Participants were randomized to receive either a single intravenous infusion of ketamine hydrochloride (0.5 mg/kg over 40 min) or placebo (0.9% saline solution) during the first session, and the converse treatment during the second session two weeks later. rsfMRI scans were obtained 2 days after each infusion. MADRS, Snaith–Hamilton Pleasure Scale (SHAPS [29]), and Temporal Experience of Pleasure Scale (TEPS) anticipatory and consummatory [30] ratings were acquired 60 min before each infusion and at 40, 80, 120, 230 min, and 1, 2, 3, 7, 10, and 11 days following each infusion. SHAPS and TEPS were used as measures of anhedonia. Two-day timepoints are analyzed here.

The primary objective was to compare the seed-to-whole brain rsFC changes of sgACC, pgACC, and dACC with ketamine *vs.* placebo, in TRD *vs.* HV. Secondary objectives were the analysis of seed-to-whole brain rsFC changes comparing ketamine *vs.* placebo in the TRD group alone, and to test whether rsFC changes correlated with ketamine’s antidepressant or anti-anhedonic effects.

### fMRI acquisition and pre-processing

Data acquisition and pre-processing were identical to those described in [19, 26]. Briefly, eight-minute rsfMRI scans (3.5 × 3.5 × 3.5 mm resolution, 64 × 64 matrix, repetition time [TR] of 2.5 s) were acquired on a 3T GE Healthcare MRI scanner (HDx; Milwaukee, WI) with an eight-channel coil. Subjects were instructed to close their eyes, relax, and remain awake. Cardiac and respiration traces were also recorded using the manufacturer’s photo-plethysmograph and respiratory belt, respectively. Pre-processing steps included de-spiking, slice-timing correction, physiological noise correction, motion alignment, blurring to 6 mm full-width-at-half-maximum, motion censoring, bandpass filtering (0.01–0.1 Hz) followed by alignment with the Montreal Neurological Institute (MNI) 152 standard space. Data points were censored if there was more than an estimated 0.2 mm of motion (Euclidean norm) per TR, and the data set was excluded from further analysis if there were more than 15 censored time points.

### Seed regions

**Fig. 1:**
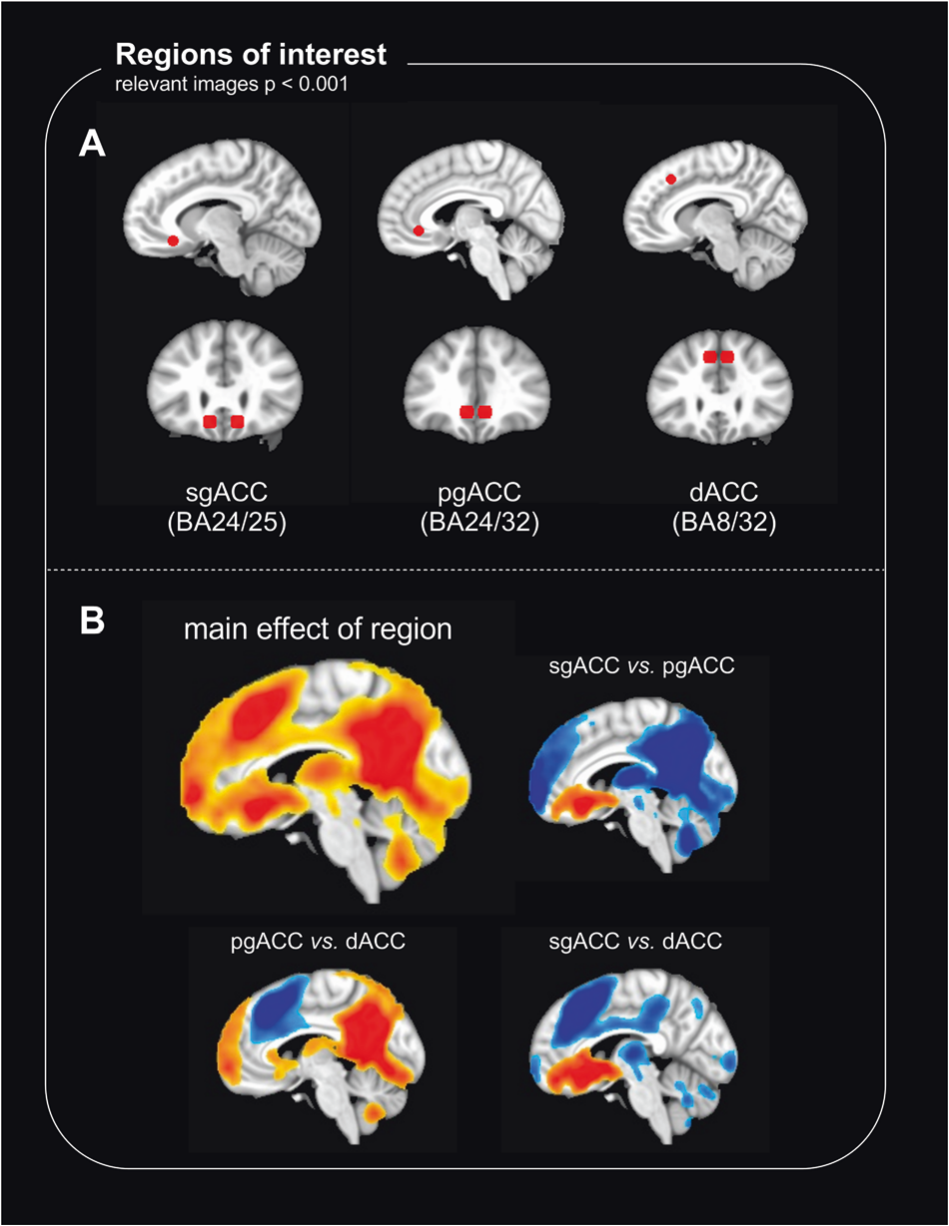
Regions of interest (ROIs) and resting-state functional connectivity (rsFC) patterns. **A** Bilateral ROI seeds for sgACC (left), pgACC (center) and dACC (right). **B** Top left shows the main effect of region in the overall statistical model, with smaller images showing contrasts between sgACC and pgACC (top right), pgACC and dACC (bottom left), and sgACC and dACC (bottom right). Warm clusters indicate higher rsFC to the former region, and cool clusters indicate higher rsFC to the latter region. Despite ROI proximity (particularly of sgACC and pgACC), there are substantial differences in rsFC to the rest of the brain.

ACC seeds were chosen based on a previous study identifying changes in subregional ACC structure and function associated with rumination in depression [31]. Left and right seeds were combined for analysis to increase signal-to-noise, as we predicted left and right seeds would show similar activity. The bilateral ACC seeds consisted of two 5 mm radius spheres and corresponded to caudal sgACC (BA24/25; MNI coordinates in RAI format: ±12 −27 −12), pgACC (BA24/32; ±6 −34 −5), and dACC (BA8/32; ±6 −28 41; Fig. 1A). Despite their proximity (particularly of sgACC and pgACC), the ROIs filled non-overlapping voxels, and showed substantial differences in rsFC (Fig. 1B). The ROIs were in regions of adequate signal (and are shown overlayed on an example EPI in Supplementary Fig. S1).

### Data analysis

Seed-to-whole-brain rsFC analyses were performed using AFNI (v 22.3.07) [32]. Due to exclusion of some scans for excess motion, the final post-ketamine sample included 26 TRD participants and 20 HVs, and the final post-placebo sample included 25 TRD participants and 17 HVs. Similar to the methodology described in [19], rsFC Fisher-transformed Z maps were generated at the single-subject level using 3dNetCorr [33].

Linear-mixed effects models (3dLMEr [34]) were used at the group-level to determine the effects of group, treatment and region on rsFC. The model took the form: group × treatment × region + order + (1|subject) + (1|subject:treatment) + (1|subject:region), where:

- group is a between-subject factor with two levels (TRD, HV);
- treatment is a within-subject factor with two levels (ketamine, placebo);
- region is a within-subject factor with three levels (sgACC, pgACC, dACC);
- group × treatment × region is the interaction between these three factors;
- order is a between-subject factor with two levels (subject’s infusion order; ketamine first or second); and
- (1|subject), (1|subject:treatment) and (1|subject:region) are a random effects within subjects.

Results of the main interaction effect (group × treatment × region) are reported as a chi-square (χ^2^) statistic with two degrees of freedom, as per the output of AFNI’s 3dLMEr. Post-hoc general linear *t*-style tests were specified to explore:

- region-specific group × treatment interactions separately for sgACC, pgACC and dACC;
- region-specific effects of treatment within the TRD group alone;
- effects of region alone, to demonstrate expected patterns of seed-whole brain rsFC; and
- group × order interaction to explore the possibility of differential expectancy and carry-over effects between the groups.

A cluster-forming threshold of *p* < 0.001 (uncorrected) with cluster-level family-wise error (FWE) correction at α < 0.05 was used to correct for multiple comparisons. Monte-Carlo simulation in AFNI (3dFWHMx and 3dClustSim) estimated a minimum cluster size of 24 voxels.

To correlate changes in connectivity with changes in symptom scores, significant clusters for the main group × treatment × region interaction, and clusters for the effect of treatment within the TRD group alone, were correlated with changes in MADRS, SHAPS and TEPS-anticipatory/TEPS-consummatory scores. Differences (ketamine – placebo) in MADRS, SHAPS and TEPS-anticipatory/TEPS-consummatory scores at day two post-infusion were correlated with changes in rsFC (ketamine – placebo) at day two post-infusion. Pearson correlation coefficients were calculated, and statistical significance was assessed at *p* < 0.05, two-tailed. Notably, SHAPS and TEPS scores were only measured in a subset of patients (*n* = 11), which reduced the sample size available for these correlations (as reported in Table 1).

Post-hoc correlation of significant clusters with symptom improvements has associated bias as described in ref. [35]; namely, the ROIs used to correlate with symptom improvements were identified in a group/condition analysis, and we analyzed the same ROIs to explore correlations with a factor that distinguishes between groups. This bias also includes ROI definition, which can include some selective noise, particularly at the periphery. Therefore, we also carried out whole-brain regressions with clinical symptom changes, which have reduced power but are not biased. To do this, we first determined whether any brain regions were differentially associated with MADRS or SHAPS scores post-ketamine *vs.* post-placebo in the TRD group, using AFNI’s 3dLMEr with the model: treatment × [MADRS/SHAPS] + order + (1|subject), where treatment is a within-subject factor with two levels (ketamine, placebo) and MADRS/SHAPS is a within-subject continuous variable.

Post-hoc general linear *t*-style tests were carried out to explore regions whose post-ketamine connectivity to the ROI was correlated with post-ketamine MADRS or SHAPS scores. To control for post-placebo connectivity, we incorporated this as a voxelwise covariate in AFNI’s 3dMVM. The model took the form [MADRS/SHAPS] + placebo_scan, where the placebo_scan was a voxelwise covariate reflecting connectivity changes under placebo, and the effect of MADRS/SHAPS is the effect of interest.

No a priori power analysis was performed because the present study was a secondary analysis of a clinical trial [27].

## Results

Participant demographic and clinical characteristics are summarized in Table 1. The significant rsFC clusters identified in the neuroimaging analyses are presented Table 2.

**Table 2:** Cluster sizes, center of mass coordinates and test statistic values of regions identified in the analyses.

Effect	Seed	Label	Size (voxels)	Peak *x*	Peak *y*	Peak *z*	Statistic
Group × treatment × region interaction (Fig. 2)	All	Right insula	28	−58.0	+9.0	+11.0	*χ*^2^ = 18.1
		Anterior vmPFC	42	+0.0	−53.0	−11.0	*χ*^2^ = 18.0
Main effect of group	All	Nil					
Main effect of treatment	All	Nil					
Main effect of region	All	See Fig. 1B					
Main effect of order	All	Nil					
Group × order interaction	All	Nil					
Group × treatment interaction (Fig. 3)	sgACC (BA24/25)	Right hippocampus and parahippocampal gyrus	29	−38.0	+30.0	−14.0	*Z* = −3.56
		Anterior vmPFC	46	+7.0	−52.0	−12.0	*Z* = 3.50
	pgACC (BA24/32)	Dorsal posterior cingulate cortex	69	+0.0	+63.0	+40.0	*Z* = −2.60
		Ventral posterior cingulate cortex	33	−1.0	+37.0	+35.0	*Z* = −2.85
	dACC (BA8/32)	Left supramarginal gyrus	26	+56.0	+22.0	+39.0	*Z* = 3.95
TRD, effect of treatment (Figs. 4 and S2)	All	Nil					
	sgACC (BA24/25)	Right hippocampus and parahippocampal gyrus	24	−40.0	+33.0	−15.0	*Z* = -3.07
		pgACC (BA24/32)	32	−2.0	−36.0	+5.0	*Z* = 2.91
		Anterior vmPFC	61	+4.0	−53.0	−12.0	*Z* = 3.79
		Right ventral striatum	37	−17.0	−2.0	−4.0	*Z* = 5.28
	pgACC (BA24/32)	Nil					
	dACC (BA8/32)	Nil					
HV, effect of treatment	All	Dorsal and ventral posterior cingulate cortex	45	+0.0	+46.0	+41.0	*Z* = 3.64
	sgACC (BA24/25)	Nil					
	pgACC (BA24/32)	Ventral posterior cingulate cortex	156	−1.0	+33.0	+35.0	*Z* = 3.87
		Right dorsal posterior cingulate cortex	34	−11.0	+65.0	+34.0	*Z* = 4.65
		Left dorsal posterior cingulate cortex	35	+15.0	+63.0	+31.0	*Z* = 4.06
	dACC (BA8/32)	Nil					
TRD, Treatment × SHAPS (Fig. 5A)	sgACC (BA24/25)	dACC	48	−2.0	−28.0	+35.0	*Z* = 5.18
Effect of SHAPS post-ketamine, controlled for placebo (Fig. 5B)	sgACC (BA24/25)	dACC	43*	−1.0	−8.0	+30.0	*t* = 3.65

Coordinates are in RAI format, in MNI space. Clusters reported at *α* < 0.05, with a cluster forming threshold of *p* < 0.001 and an extent of 24 voxels, except *reported at a threshold of *p* < 0.005.

### Clinical results

At day two following treatment, ketamine significantly improved MADRS scores (mean difference -6.5 [95% CI -10.6 to -2.5]; paired *t*-test, *p* = 0.003), TEPS-anticipatory scores (mean difference 6.8 [95% CI 2.9 to 9.5]; paired *t*-test, *p* = 0.038), TEPS-consummatory scores (mean difference 5.6 [95% CI 1.8 to 6.1]; paired *t*-test, *p* = 0.018) and showed a trend to improve SHAPS scores (mean difference -5.2 [95% CI -10.7 to 0.2]; paired *t*-test, *p* = 0.057) compared to placebo (Supplementary Fig. S2). There was no correlation between the improvement in MADRS scores and the improvement in SHAPS (*R*^2^ = 0.169, *p* = 0.173) or TEPS-consummatory (*R*^2^ = 0.034, *p* = 0.544) scores. There was correlation between the improvement in TEPS-anticipatory and MADRS (*R*^2^ = 0.371, *p* = 0.027) scores. This suggests the depression and anhedonia scores are measuring distinct but partially overlapping constructs.

### Ketamine differentially modulates ACC subregional rsFC compared to placebo in TRD *vs.* HV

**Fig. 2:**
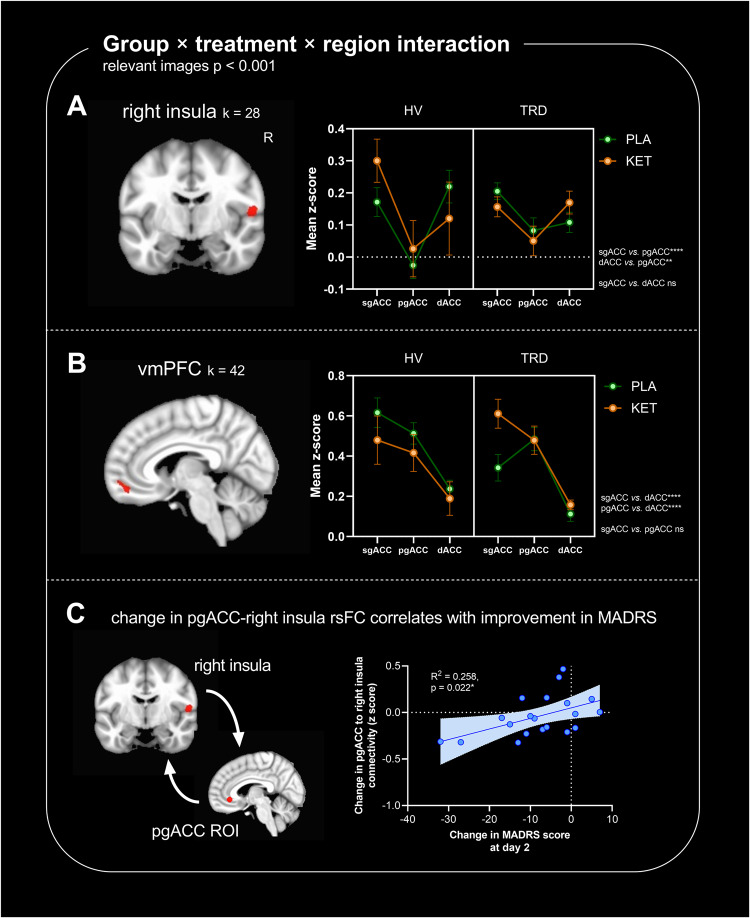
Clusters showing a significant group × treatment × region interaction. Images show significant clusters from a seed-based resting-state functional connectivity (rsFC) analysis, cluster thresholded at *α* < 0.05, with a cluster forming threshold of *p* < 0.001 and an extent of 24 voxels. Graphs show mean connectivity values (Fisher’s *z*-scores) between the seed and extracted cluster from whole brain analysis. HV healthy volunteers (*n* = 21), TRD treatment-resistant depression (*n* = 29), PLA placebo, KET ketamine. *p < 0.05, **p < 0.01, ***p < 0.001, ****p < 0.0001, ns not significant. **A** rsFC of ACC subregions to the right insula was differentially modulated by ketamine *vs.* placebo, in TRD *vs.* HV (group × treatment × region interaction, *F*_2,105_ = 7.40, *p* = 0.001). sgACC and dACC showed similar rsFC patterns to the right insula (*p* = 0.229) whereas sgACC *vs.* pgACC (*p* < 0.0001) and dACC *vs.* pgACC (*p* = 0.001) showed differences. **B** rsFC of ACC subregions to the anterior ventromedial prefrontal cortex (vmPFC; BA10m) was also differentially modulated across treatment and group (group × treatment × region interaction, *F*_2,105_ = 4.64, *p* = 0.012). Here, sgACC and pgACC showed similar rsFC patterns (*p* = 0.636); dACC showed distinct rsFC to the anterior vmPFC compared to sgACC (*p* < 0.0001) and pgACC (*p* < 0.0001). **C** In the 20 TRD participants who had both ketamine and placebo scan data, [ketamine – placebo] changes in rsFC between pgACC and the right insula correlated with [ketamine – placebo] improvements in Montogomery-Åsberg Depression Rating Scale (MADRS) scores at day 2 post-infusion (*R*^2^ = 0.258, *p* = 0.022).

At day two following treatment, fMRI imaging revealed a significant group × treatment × region interaction, suggesting differential modulation by ketamine compared to placebo, in TRD *vs.* HV, across the three ACC subregions. Significant interaction clusters included the right insula (Fig. 2A) and bilateral anterior vmPFC (BA10m; Fig. 2B). Changes in rsFC between pgACC – but not sgACC or dACC – and the right insula correlated with improvements in MADRS scores (Fig. 2C). rsFC changes did not correlate with improvements in SHAPS scores (not shown).

**Fig. 3:**
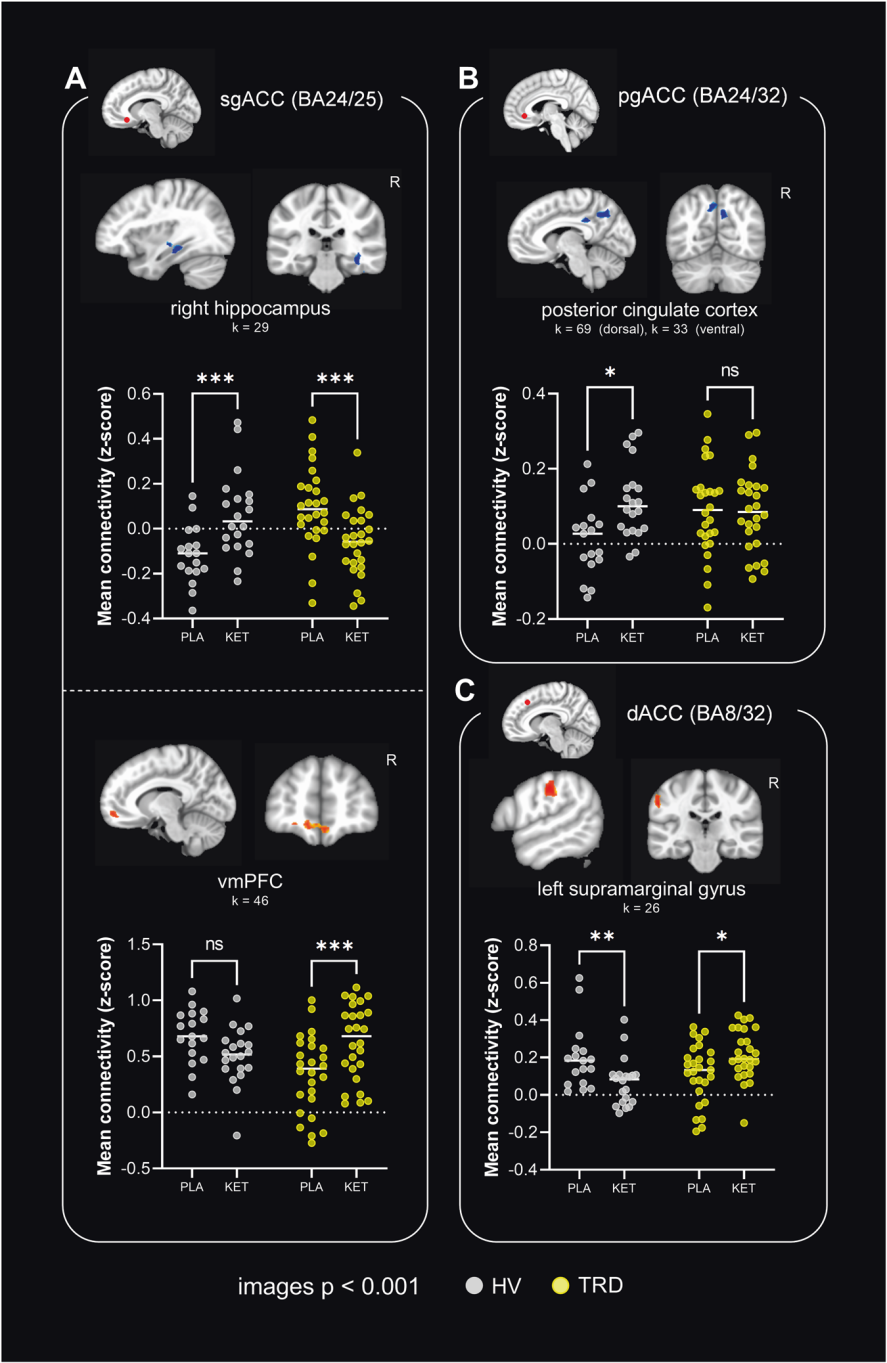
Differential effects of ketamine *vs.* placebo in TRD (*n* = 29 total) *vs.* HV (*n* = 21 total) in individual ACC subregions. Images show significant clusters from seed-based resting-state functional connectivity (rsFC) analysis, cluster thresholded at *α* < 0.05, with a cluster forming threshold of *p* < 0.001 and an extent of 24 voxels. Graphs show mean rsFC values (Fisher’s z-scores) between the seed and extracted cluster from whole brain analysis. HV healthy volunteers, TRD treatment-resistant depression, PLA placebo, KET ketamine. *p < 0.05, **p < 0.01, ***p < 0.001, ****p < 0.0001, ns not significant. **A** sgACC-right hippocampal rsFC (treatment × group, *F*_1,35_ = 30.7, *p* < 0.0001) and sgACC-anterior ventromedial prefrontal cortex (vmPFC; BA10m) rsFC (treatment × group, *F*_1,35_ = 20.1, *p* < 0.001) was differentially altered by ketamine *vs.* placebo in TRD *vs.* HV. In the former case, rsFC was increased by ketamine in HV (effect of treatment: *p* < 0.001) but decreased in TRD (*p* < 0.001). In the latter case, rsFC was increased by ketamine in TRD (*p* < 0.001) with a tendency to be decreased in HV (*p* = 0.065). **B** pgACC-posterior cingulate cortex rsFC was differentially altered by ketamine *vs.* placebo in TRD *vs.* HV (larger dorsal cluster: treatment × group, *F*_1,35_ = 5.12, *p* = 0.029). Whilst ketamine significantly increased rsFC in HV (effect of treatment: *p* = 0.013) there was no significant change in the TRD group (*p* = 0.994). **C** dACC-left supramarginal gyrus rsFC was differentially altered by ketamine *vs.* placebo in TRD *vs.* HV (treatment × group, *F*_1,35_ = 21.6, *p* < 0.0001). Ketamine decreased rsFC in HV (effect of treatment: *p* = 0.002), but increased connectivity in TRD (*p* = 0.011).

Post-hoc general-linear *t*-style tests for each ACC subregion revealed significant group × treatment interactions between sgACC and the anterior vmPFC & right hippocampus (Fig. 3A); pgACC and the posterior cingulate cortex (Fig. 3B); and dACC and the supramarginal gyrus (Fig. 3C).

### In TRD, changes in sgACC (BA24/25) rsFC correlate with improvements in anhedonia scores

**Fig. 4:**
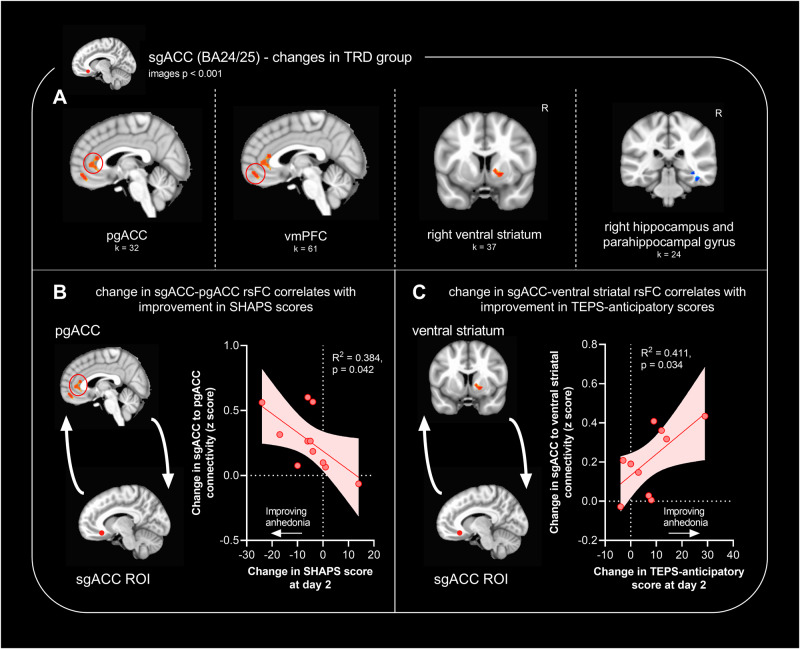
Modulation of sgACC (BA24/25) connectivity by ketamine *vs.* placebo, in patients with TRD (*n* = 26 post-ketamine and *n* = 25 post-placebo; *n* = 11 for symptom correlations). Images show significant clusters from seed-based resting-state functional connectivity (rsFC) analysis, cluster-thresholded at α < 0.05, with a cluster forming threshold of *p* < 0.001 and an extent of 24 voxels. **A** In patients with TRD, ketamine increased sgACC-pgACC, anterior vmPFC and ventral striatal rsFC compared to placebo, but decreased sgACC-hippocampal formation rsFC. **B** [Ketamine – placebo] increases in rsFC between sgACC-pgACC correlated with improvements in Snaith Hamilton Pleasure Scale (SHAPS) scores (*R*^2^ = 0.384, *p* = 0.042). **C** [Ketamine – placebo] increases in rsFC between sgACC-ventral striatum correlated with improvements in Temporal Experience of Pleasure Scale (TEPS)-anticipatory scores (*R*^2^ = 0.411, *p* = 0.034; note that one point is obscured beneath another on the graph) but not TEPS-consummatory scores (*R*^2^ = 0.137, *p* = 0.263; not shown).

Following the identification of significant interaction effects, general linear *t*-style tests were carried out to explore the effect of treatment (ketamine *vs.* placebo) within the TRD group alone (*n* = 26 post-ketamine and *n* = 25 post-placebo) for rsFC of sgACC, pgACC and dACC ROIs separately. No significant clusters corresponding to an effect of treatment in the TRD group were found for pgACC or dACC rsFC. However, sgACC rsFC was significantly modulated by ketamine in the TRD group: connectivity was increased to bilateral pgACC, bilateral anterior vmPFC, and right ventral striatum, and decreased to right hippocampus/parahippocampal gyrus (Fig. 4A).

SHAPS scores and TEPS-anticipatory/TEPS-consummatory scores, together with fMRI data for both ketamine and placebo scans, were available for a subset of TRD patients (*n* = 11). We correlated [ketamine – placebo] changes in SHAPS/TEPS scores with the [ketamine – placebo] changes in sgACC rsFC to the pgACC, vmPFC, striatal and hippocampal clusters identified above. For these patients, ketamine-induced increases in sgACC-pgACC rsFC correlated with improvements in SHAPS scores (Fig. 4B). Changes to sgACC-ventral striatal rsFC were significantly correlated with improvements in TEPS-anticipatory (*R*^2^ = 0.411, *p* = 0.034; Fig. 4C) and not TEPS-consummatory (*R*^2^ = 0.137, *p* = 0.263) scores (and showed a trend to correlate with improvements in SHAPS scores, *R*^2^ = 0.287, *p* = 0.082). This suggests a differential contribution of sgACC-striatal rsFC to temporally distinct types of anhedonia symptoms. Changes to sgACC-hippocampal rsFC showed a trend to correlate with improvements in SHAPS scores (*R*^2^ = 0.338, *p* = 0.061) and changes to sgACC-anterior vmPFC rsFC did not correlate. Changes in sgACC rsFC did not correlate with improvements in MADRS scores (*n* = 20; not shown).

### Whole-brain regressions of clinical scores with sgACC connectivity reveal correlations between sgACC-dACC rsFC and anhedonia scores

**Fig. 5:**
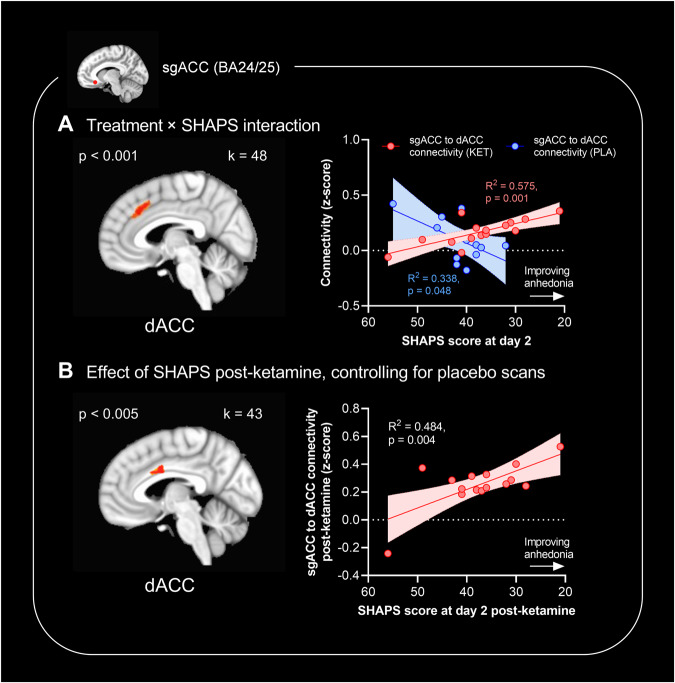
Whole-brain regressions of anhedonia symptom scores (Snaith Hamilton Pleasure Scale, SHAPS) with sgACC rsFC changes in patients with TRD (*n* = 15 post-ketamine and *n* = 12 post-placebo). For figure **A**, the image shows significant clusters from seed-based resting-state functional connectivity (rsFC) analysis, cluster-thresholded at *α* < 0.05, with a cluster forming threshold of *p* < 0.001 and an extent of 24 voxels. For figure **B**, the image shows a dACC cluster with a threshold of *p* < 0.005. Graphs show correlations between absolute SHAPS scores *vs.* absolute sgACC-dACC rsFC (Fisher’s z-score; either ketamine and placebo [**A**] or ketamine alone [**B**]) measured two days following infusion. **A** sgACC-dACC rsFC was positively associated with SHAPS scores post-ketamine (*R*^2^ = 0.575, *p* = 0.001) but was negatively associated with SHAPS scores post-placebo (*R*^2^ = 0.338, *p* = 0.048). **B** After controlling for rsFC changes post-placebo, a 39-voxel dACC cluster was significant at *p* < 0.005 voxels and sgACC rsFC to this cluster was correlated with post-ketamine SHAPS scores (*R*^2^ = 0.484, *p* = 0.004).

Post-hoc correlation of significant clusters with symptom improvements has associated bias (as described in “Methods” section (Data Analysis)); a regression analysis at the whole-brain level has reduced power but is unbiased. We carried out whole-brain regressions of SHAPS/MADRS scores with sgACC rsFC maps, firstly exploring whether any regions showed significantly different relationships with clinical scores following ketamine *vs.* placebo (treatment × score interaction). In 15 TRD patients where fMRI and SHAPS data were available post-ketamine, analysis revealed a cluster in the dACC (BA24/32, Fig. 5A) whose rsFC to sgACC was positively associated with post-ketamine SHAPS scores but negatively associated with post-placebo SHAPS scores. No significant regions were found associated with MADRS scores (not shown).

We additionally explored whether any clusters showed a significant association with SHAPS/MADRS scores following ketamine treatment, after controlling for connectivity changes under placebo by incorporating placebo connectivity as a voxelwise covariate. Whilst no clusters were identified at *p* < 0.001, at a relaxed uncorrected threshold of *p* < 0.005, we noted a single dACC cluster at *p* < 0.005, k = 39 voxels, whose rsFC to sgACC was positively associated with post-ketamine SHAPS scores (Fig. 5B). No significant regions were found associated with post-ketamine MADRS scores (not shown).

## Discussion

Here we show that subregions of the ACC undergo differential modulation by intravenous ketamine, compared to placebo, in patients with TRD *vs.* HV. Ketamine differentially modulates ACC subregional rsFC to the right insula and anterior vmPFC in TRD *vs.* HV. rsFC changes between pgACC and the insula correlated with improvements in MADRS scores, supporting suggestions that ketamine’s modulation of pgACC connectivity is important in its post-acute (in this study, day two) antidepressant effects [4].

Post-hoc examination of rsFC changes within each ACC subregion separately highlighted that rsFC of ventral ACC divisions (pgACC and sgACC) to affective and DMN regions (posterior cingulate cortex and anterior vmPFC/hippocampus respectively) was modulated. By contrast, ketamine modulated connectivity of dACC to the supramarginal gyrus, which constitutes part of the somatosensory association cortex. These differential effects are consistent with known connectivity of the ACC: ventral regions contribute to affective networks and the DMN, whereas dorsal regions contribute to frontoparietal somatosensory and attention networks [6].

In TRD patients, sgACC underwent the most substantial rsFC changes of ACC subregions in response to ketamine compared to placebo. Whilst post-hoc comparisons identified that ketamine significantly altered sgACC rsFC to pgACC, hippocampus/parahippocampal gyrus, ventral striatum, and anterior vmPFC, no significant changes were found for pgACC or dACC. We previously hypothesized that ketamine may change sgACC connectivity to nodes of the DMN [4] and we found evidence to support this: ketamine decreased sgACC-hippocampal formation rsFC and increased sgACC-pgACC rsFC. Importantly, increases in sgACC-pgACC rsFC correlated with improvements in SHAPS scores, suggesting that ketamine’s anti-anhedonic effects are mediated by changes in sgACC-pgACC connectivity.

Connectivity changes between pgACC and sgACC have previously been implicated in MDD. Anatomically, these regions are densely interconnected, with pgACC/32 making excitatory contacts on sgACC/25 pyramidal cells as well as inhibitory interneurons [22, 36]. Higher cortical regions such as the dorsolateral PFC and pgACC/32 are thought to provide top-down regulation of sgACC/25, and the loss of this in MDD is thought to impair adaptive coping [21]. In this study, ketamine increased connectivity between pgACC and sgACC, which may reflect an amelioration of impairments in top-down regulation. Here we show this may have clinical relevance; pgACC/sgACC rsFC change correlated with improvements in SHAPS scores.

Connectivity between the ACC and the hippocampus has also been consistently implicated in depression, as these regions are particularly sensitive to the effects of chronic stress [37, 38]. Hippocampal-ACC circuitry has been implicated in the antidepressant effects of ketamine. In rodents, optogenetic and pharmacogenetic activation of hippocampal-infralimbic projections replicates the sustained antidepressant effects of ketamine [39]. Several human neuroimaging studies also support ketamine-induced modulations of hippocampal-prefrontal circuitry as being relevant to its antidepressant effects [20, 40, 41]. In this study, sgACC-hippocampal rsFC was decreased by ketamine compared to placebo; such changes may be relevant to autobiographical recall, rumination [42], and the regulation of downstream structures important in emotion, such as the amygdala [43].

Notably, sgACC-ventral striatal connectivity was significantly altered by ketamine. sgACC-ventral striatal rsFC changes correlated with improvements in TEPS-anticipatory scores but not TEPS-consummatory scores, suggesting differential involvement of these prefrontal-striatal circuits in temporally distinct aspects of anhedonia. This finding back-translates to preclinical primate work: connectivity between sgACC and ventral regions of the striatum has been causally implicated in ketamine’s ability to ameliorate anticipatory (but not consummatory) reward processing deficits in marmoset models [18]. This finding also highlights the importance of distinguishing between different subtypes of anhedonic symptoms; for example, the SHAPS scale does not distinguish between anticipatory *vs.* consummatory anhedonia, and so may be insensitive to changes in specific symptom clusters.

When we carried out whole-brain regressions exploring sgACC rsFC changes associated with ketamine’s anti-anhedonic effects, we identified a cluster in the dACC where connectivity was positively associated with lower anhedonia as measured by SHAPS scores. The dACC is known to be interconnected with sgACC [22] and changes in dACC activity have previously been associated with ketamine’s anti-anhedonic effects in both preclinical marmoset models [17] and in humans [1, 2]. Increased connectivity between sgACC and dACC may reflect increased connectivity of sgACC to nodes of the salience network, important in reward processing by identifying salient reward-predicting stimuli [44]. In sum, these data suggest that sgACC connectivity to a network including other ACC subregions and the ventral striatum may be modulated by ketamine to improve anhedonia. It is important to note, however, that from fMRI data alone, we cannot be sure if these correlations reflect causal relationships, nor can we establish whether rsFC changes reflect changes in excitatory *vs.* inhibitory connections between these regions.

Limitations of this study include, firstly, the small sample size (particularly when exploring correlations between changes in rsFC and anhedonia scores) which may limit the power to detect changes in ACC rsFC and associations with improvements in symptoms. The within-subject crossover design mitigates this issue to a degree, but replication of these findings in trials with larger sample sizes is critical. Second, the sample size was too small to perform cross-validation using a leave-one-out framework, which would otherwise handle potential bias from the inferential tests being performed on clusters taken from the analysis. Third, the fixed time point analyzed in this study (two days), means we could not ascertain how changes in rsFC develop over the duration of ketamine’s antidepressant effects. Finally, whilst the integrity of the blind for score raters was assessed as reported in a prior manuscript [27], the integrity of the blind for participants was not assessed; unblinding effects may confound the improvements in clinical scores following ketamine.

## Conclusion

We show that different subregions of the ACC are differentially modulated by ketamine, compared to placebo, in TRD *vs.* HV. rsFC between the ACC, insula, and anterior vmPFC is modulated by ketamine compared to placebo, and changes in pgACC-right insula connectivity correlate with improvements in MADRS scores. Post-hoc comparisons within the TRD group illustrate that sgACC undergoes the most substantial modulations, with changes in sgACC-pgACC, sgACC-ventral striatal, and sgACC-dACC connectivity correlating with anhedonia scores. These data provide preliminary evidence suggesting ketamine’s modulation of ACC subregional rsFC is important in mediating its antidepressant and anti-anhedonic effects, and illustrate the importance of ACC segmentation in understanding ketamine’s effects.

### Supplementary information

Supplemental

## Data Availability

Data are available on request from the corresponding author.

## Code availability

Code is available on request from the corresponding author.
