# Supplementary material for: Preliminary evidence that ketamine alters anterior cingulate resting-state functional connectivity in depressed individuals
Source: Transl Psychiatry. 2023 Dec 1;13:371. doi: 10.1038/s41398-023-02674-1 (PMC10692230; doi:10.1038/s41398-023-02674-1)
Supplement: Supplementary file 1 — Supplemental [file 41398_2023_2674_MOESM1_ESM.docx]

# Supplementary materials

## Supplementary methods

### Participant Information

Participants were evaluated using the Structured Clinical Interview for Axis 1 DSM-IV Disorders (SCID)-patient (First et al., 2002a) and nonpatient (NP) (First et al., 2002b) versions. HVs had no family history of Axis I disorders in first-degree relatives as determined by the SCID-NP. All participants were deemed to be in good physical health, as determined by medical history, physical examination, blood tests, chest x-ray, electrocardiogram, toxicology, and urinalysis.

### Resting-state functional magnetic resonance imaging (rsfMRI) acquisition and pre-processing

rsfMRI scans were obtained two days following each infusion. Ketamine had been fully metabolized by this time point, so neural effects identified at imaging are not attributable to ketamine’s immediate pharmacological effects.

Data acquisition and preprocessing were identical to (Evans et al., 2018; Mkrtchian et al., 2019). High-resolution structural images were obtained using a T1-weighted 3D fast spoiled gradient recalled echo (FSPGR) sequence with an 8.8s repetition time (TR), 3.4ms echo time (TE), 450ms inversion recovery time (IR), 13 degrees flip angle (FA), and with a 1mm isotropic resolution. Whole-brain rsfMRI images were obtained using a gradient recalled echo planar image (EPI) sequence with a 90˚ flip angle, 192 volumes, 45 slices per volume, 3.5 x 3.5 x 3.5 mm resolution, 64 x 64 matrix, TR of 2.5 s, TE of 25 ms, anterior-posterior phase encoding direction and interleaved acquisition. In addition, cardiac and respiration traces were recorded during each scan using the GE photoplethysmograph and respiratory belt.

Preprocessing was accomplished in AFNI with the afni_proc script, including despiking, slice-time correction, nuisance signal regression (motion: 12-parameter affine, registered to the third volume; physiological: slice-based, generated with McRetroTS), 6mm full-width-at-half-maximum (FHWM) spatial smoothing, band-pass filtering (0.01-0.1Hz), alignment to the Montreal Neurological Institute (MNI) 152 standard space, and motion censoring. Alignment to standard space was achieved by first aligning the structural image to the EPI with an affine transform using the LPC cost-function (align_epi_anat.py in AFNI). The anatomical image was non-linearly warped to the MNI 152 standard template and the EPI was transformed to standard space using the concatenated transformation matrices produced from the anatomical alignment steps. Image sequences were censored if there was movement greater than 0.2 mm (Euclidean norm) per TR. If there were more than 15 censored time points per dataset, the dataset was excluded from further analysis. Motion (de-meaned and derivative) regressors were removed from the original time series simultaneously with band-pass filtering.

### fMRI data information

As reported previously (Evans et al., 2018; Mkrtchian et al., 2019), data from specific scans were excluded for the following reasons: incomplete physiological data; excessive motion (>0.2mm/TR); and high correlation between the respiration volume trace and the average global signal, which increased correlations across the brain. Functional connectivity analyses were conducted on 20 post-ketamine and 17 post-placebo HV scans and on 26 post-ketamine and 25 post-placebo TRD scans. Regions of interest (ROIs) were in areas of adequate signal, as shown in the signal map overlay in **Supplementary Figure S1**.

Linear mixed-effects models were conducted on the rsfMRI scans obtained two days post-infusion. This analysis was chosen due to its advantages in analyzing mixed design data (between- and within-subjects) and because it allowed us to retain participants with only one post-infusion scan, thus increasing statistical power (Chen et al., 2013). See main methods (**Data analysis**) for a description of the statistical analyses.

### Clinical effects of ketamine

Treatment effects of ketamine *vs.* placebo on Montgomery-Åsberg Depression Rating Scale (MADRS), Snaith-Hamilton Pleasure Scale (SHAPS), and Temporal Experience of Pleasure Scale (TEPS)-anticipatory/consummatory scores taken at two days after infusion (the same timepoint as fMRI imaging) can be found in **Supplementary Figure S2**.

## Supplementary references

Chen, G., Saad, Z. S., Britton, J. C., Pine, D. S., & Cox, R. W. (2013). Linear mixed-effects modeling approach to FMRI group analysis. *NeuroImage*, *73*, 176–190. https://doi.org/10.1016/j.neuroimage.2013.01.047

Evans, J. W., Szczepanik, J., Brutsché, N., Park, L. T., Nugent, A. C., & Zarate, C. A. (2018). Default Mode Connectivity in Major Depressive Disorder Measured Up to 10 Days After Ketamine Administration. *Biological Psychiatry*, *84*(8), 582–590. https://doi.org/10.1016/j.biopsych.2018.01.027

First, M., Spitzer, R., Gibbon, M., & Williams, J. (2002a). Structured Clinical Interview for DSM-IV-TR Axis I Disorders, Research Version, Non-Patient Edition (SCID-I/NP). In *Biometrics Research*. New York State Psychiatric Institute.

First, M., Spitzer, R., Gibbon, M., & Williams, J. (2002b). Structured Clinical Interview for DSM-IV-TR Axis I Disorders, Research Version, Patient Edition (SCID-I/P). In *Biometrics Research*. New York State Psychiatric Institute.

Mkrtchian, A., Lally, N., Nugent, A., Evans, J., Roiser, J., & Zarate, C. (2019). The effect of ketamine on reinforcement learning in treatment-resistant depression. *Neuropsychopharmacology*, *44*, 456–457. https://doi.org/10.1038/s41386-019-0547-9

## Supplementary figures and legends

| 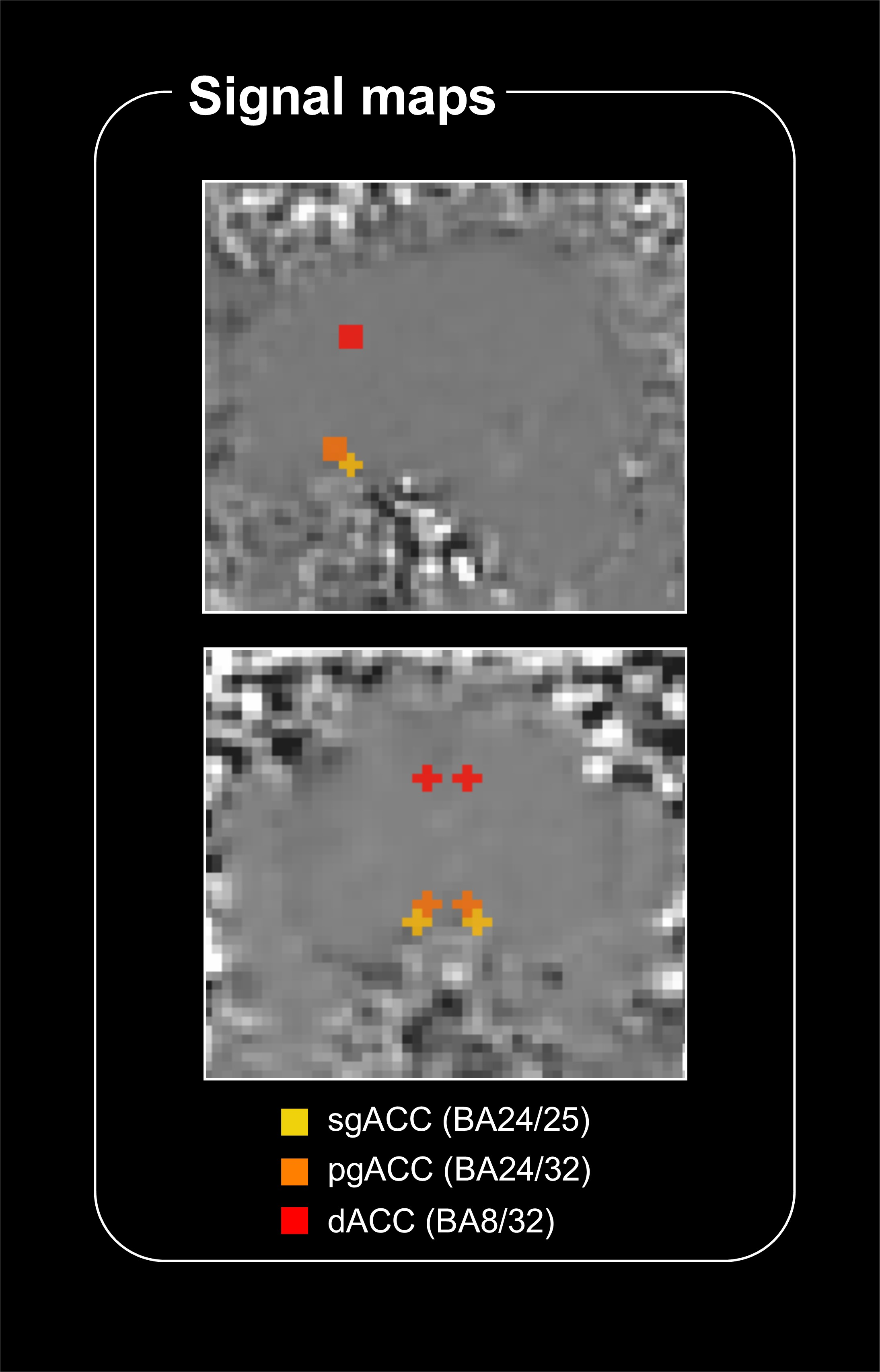 |
| --- |
| **Figure S1.** Signal maps showing regions of interest (ROIs) overlayed onto an exemplar echoplanar image, indicating ROIs are in areas of adequate signal. The sgACC and pgACC ROIs are close together but occupy non-overlapping voxels. |

| 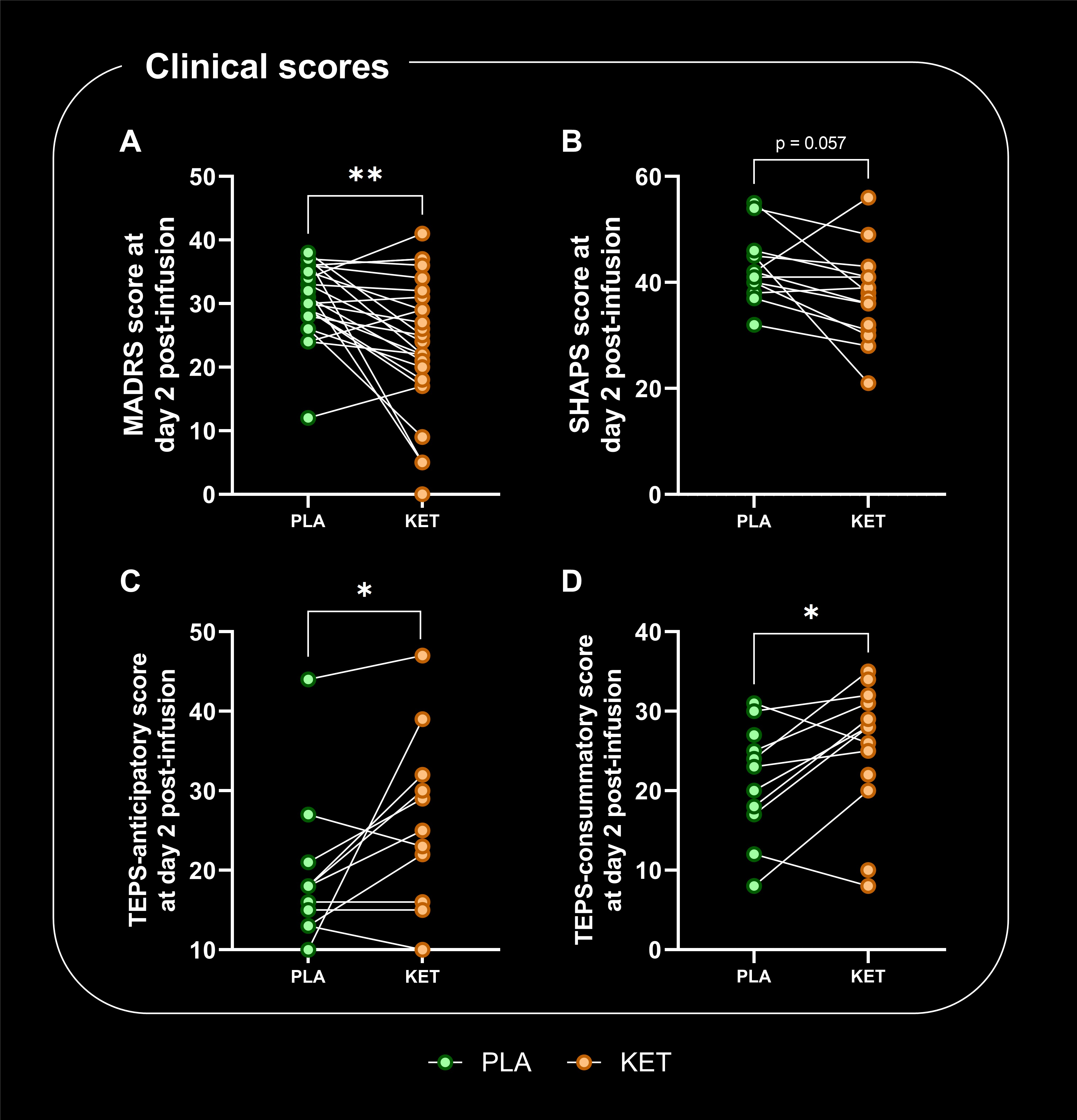 |
| --- |
| **Figure S2**. **Clinical scores measured two days after placebo or ketamine.** PLA = placebo, KET = ketamine. MADRS, n = 24; other scores, n = 13. **A** Ketamine significantly improved Montogomery-Åsberg Depression Rating Scale (MADRS) scores compared to placebo (paired *t*-test, p = 0.002). **B** Ketamine showed a trend effect to improve Snaith Hamilton Pleasure Scale (SHAPS) scores compared to placebo (paired *t*-test, p = 0.057). **C** Ketamine significantly improved Temporal Experience of Pleasure Scale (TEPS)-anticipatory scores compared to placebo (paired *t*-test, p = 0.038). **D** Ketamine significantly improved TEPS-consummatory scores compared to placebo (paired *t*-test, p = 0.018). |
